# The Role of *Setophoma terrestris* in Pink Root Disease: New Insights and Host Range in Brazil

**DOI:** 10.3390/jof11080581

**Published:** 2025-08-05

**Authors:** Gustavo Henrique Silva Peixoto, Thais Franca Silva, Laura Freitas Copati, Ailton Reis, Valter Rodrigues Oliveira, Valdir Lourenço, Danilo Batista Pinho

**Affiliations:** 1 Universidade de Brasília, Departamento de Fitopatologia, Brasília 70910-900, Brazil; gugspeixoto@gmail.com (G.H.S.P.); thais.franca08@gmail.com (T.F.S.); laura.freitas@aluno.unb.br (L.F.C.); 2 Embrapa Hortaliças, Brasília 72305-970, Brazil; valter.oliveira@embrapa.br (V.R.O.); valdir.lourenco@embrapa.br (V.L.J.)

**Keywords:** diagnosis, Alliaceae, *Pyrenochaeta*, *Phoma*, taxonomy

## Abstract

The soil-borne fungi, *Setophoma terrestris* and *Fusarium* spp., are often associated with pink root, although the etiology of the disease remains doubtful. While recognized as the primary inoculum, studies show conflicting views on the formation of chlamydospores and microsclerotia in *Setophoma*. Therefore, this study aims to clarify the etiology of the pink root of garlic and onion and the formation of chlamydospores and microsclerotia in *Setophoma*. The isolates were obtained from symptomatic tissues of garlic, leeks, brachiaria, onions, chives, and maize collected from seven different states in Brazil. Representative isolates were selected for pathogenicity tests. Sequence comparison of the tubulin gene showed *Setophoma* (*n* = 50) and *Fusarium* clades (*n* = 25). Garlic and onion plants inoculated with *Setophoma* showed pink root symptoms, while plants inoculated with different *Fusarium* isolates remained asymptomatic. Multigene analysis of pathogenic isolates confirms that only *Setophoma terrestris* causes pink root in garlic and onion. In addition, brachiaria, chives, and leeks are newly identified hosts of this pathogen in Brazil. To our knowledge, the main sources of primary inoculum of the disease are chlamydospores, pycnidia, colonized roots of garlic, onion, and plant debris of susceptible crops. The new information obtained in this study will be fundamental for researchers in the development of genotypes that are resistant to pink root and will help the efficient management of the disease.

## 1. Introduction

China and India are the world’s largest producers of garlic and onions. In 2022, Brazil was the 11th and 13th largest world producer of onion (1.65 million tons) and garlic (181.1 thousand tons), respectively [1]. Due to the insufficiency of production to satisfy national demand, Brazil regularly imports from other countries. The main obstacle to expanding garlic and onion cultivation is the high cost of production, primarily due to the occurrence of diseases [2].

Pink root disease is the main disease affecting garlic and onions during periods of drought stress and high temperatures [3,4]. It is characterized by an initial light pink discoloration of infected roots, which gradually become shortened, water-soaked, and dark purple before collapsing and dying. The continuous infection of newly formed roots compromises nutrient and water uptake, leading to stunted growth, leaf etiolation and wilting, and ultimately the formation of undersized, poor-quality bulbs with reduced storage potential [3]. Implementing control measures against the pathogen can enhance production by as much as 70% [5,6].

This disease was first documented in the state of Texas, United States, in 1917 [7]. In Brazil, the disease was first reported affecting onions in the Zona da Mata Mineira region in 1960 [8]. Subsequently, it was also found in the states of Rio Grande do Sul and São Paulo, and currently occurs in all garlic- and onion-producing regions [2,9,10]. Despite reports of the pink root causal agent in China, it is listed as a quarantine pest by the Ministry of Agriculture and Rural Affairs of the People’s Republic of China [11,12].

The first causal agent of the disease was proposed as *Fusarium malli* [13]. Later, other *Fusarium* species were associated with pink root [14,15,16,17]. The immersion of roots in a solution of mercuric chloride (500:1) for 3 min revealed the presence of *Phoma* sp. [18]. Besides the isolation approach, pathogenicity tests revealed that *Phoma terrestris* is the causal agent of pink root, while *Fusarium* species were associated with rotten roots [18,19]. The simultaneous inoculation of both pathogens confirmed the opportunistic nature of *Fusarium* species, which failed to induce pink root symptoms [19]. Nevertheless, *Fusarium* species are often associated with pink root [17,20,21,22,23,24].

The frequent recovery of isolates that form pycnidia containing setae indicated *P. terrestris* as the main etiological agent of pink root [25]. Based on the presence of setae on the pycnidium, *P. terrestris* was transferred to *Pyrenochaeta terrestris* (Hansen) Gorenz [26]. Later, based on phylogenetic analysis, this taxon was reclassified as *Setophoma terrestris*. This species is characterized by the presence of setose pycnidia, phialidic conidiogenic cells, and ellipsoidal to subcylindrical, aseptate, and hyaline conidia [27].

Although the taxonomic studies on *Setophoma* do not mention the presence of chlamydospores and microsclerotia, these survival structures are considered the primary inoculum source of pink root [3,17,28]. Since it is uncertain whether species of *Setophoma* and *Fusarium* could be associated with pink root, especially considering that most identifications are based on only a few isolates or limited to the examination of morphological data, it is crucial to characterize fungal isolates obtained from plant hosts exhibiting typical symptoms of the disease.

Furthermore, it has been shown that a molecular perspective, combined with morphological data, is required to resolve plant pathogen species complexes. This combined approach has proven effective in revealing previously uncharacterized species affecting different crops [27,29]. For example, molecular characterization of *Setophoma* isolates on tea plants revealed a total of four new species [29]. Although molecular characterization studies involving a large number of *Setophoma* isolates from garlic and onion are still lacking, various molecular diagnostic methods have been developed for the detection of this pathogen [11,17,30].

In Brazil, the frequent association of *Fusarium* with pink root symptoms has led growers to believe that this fungus may also be involved in the etiology of the disease. Therefore, this study aims to clarify the etiology of the pink root of garlic and onion and the formation of chlamydospores and microsclerotia in *Setophoma*.

## 2. Materials and Methods

### 2.1. Obtaining and Preserving Isolates

Root samples (*n* = 75) were obtained in garlic, onion, leeks, brachiaria, chives, and maize from 17 cities in the Brazilian states of Distrito Federal, Goiás, Minas Gerais, São Paulo, Santa Catarina, Paraná, and Bahia. Fungal isolation in pure culture was carried out using the direct method [31], by depositing conidia onto potato dextrose agar (PDA) medium (200 g/L potato infusion, 25 g/L agar, and 20 g/L dextrose), from garlic, leeks, brachiaria, onions, chives, and maize showing typical pink root symptoms and previously surface-disinfected with 70% alcohol. After 7 days, pure cultures were established by transferring a fragment of a hyphal tip to a new Petri dish containing PDA. The isolates (Supplementary Table S1) were stored at Coleção de Culturas Fúngicas da UnB (CCUB, Universidade de Brasília, Brasília, Brazil) in 2 mL microtubes by the Castellani method and glycerol 10% (*v*/*v*) in −80 °C ultrafreezer.

### 2.2. Total DNA Extraction

The fungal isolates were grown on Erlenmeyer flasks containing Potato Dextrose Broth (PDB; 200 g/L potato infusion and 20 g/L dextrose) medium, at 25 °C for 7 days, with a 12 h photoperiod. The mycelium (100 µg) was collected using a sterile toothpick and deposited in 1.5 mL microtubes containing 20 µL of Tris-EDTA (TE) buffer. The total DNA extraction was performed using the Wizard Genomic Purification Kit (Promega^®^, Madison, WI, USA) [32]. The presence and quality of total DNA were analyzed on 1% agarose gel electrophoresis with a 1 Kb ladder, stained with GelRed (Biotium^®^, Fremont, CA, USA), and visualized under UV light. The DNA samples were stored at −20 °C.

### 2.3. Amplification and Sequencing

Partial sequences of the gene *β-tubulin* (*tub*) were amplified using the primer set T1 (5′-AACATGCGTGAGATTGTAAGT-3′) and T22 (5′-TCTGGATGTTGTTGGGAATCC-3′) [33]. This genomic region was used to make an initial identification of 75 isolates using BLASTN similarity scores. Twelve isolates of different clades and locations identified based on *tub* sequence data were selected to assign definitive species demarcations for *Setophoma* isolates. Then, partial nucleotide sequences of the four following genomic regions were sequenced: *large subunit of ribosomal DNA* (*lsu-its*), *translation elongation factor* (*tef*), *glyceraldehyde-3-phosphate dehydrogenase* (*gapdh*), and *RNA polymerase subunit II* (*rpb2*), using the primer pairs V9G (5′-TTACGTCCCTGCCCTTTGTA-3′) and LR5 (5′-TCCTGAGGGAAACTTCG-3′) [34,35], EF1F (5′-TGCGGTGGTATCGACAAGCGT-3′) and EF2R (5′-AGCATGTTGTCGCCGTTGAAG-3′) [36], GPD1 (5′-CAACGGCTTCGGTCGCATTG-3′) and GPD2 (5′-GCCAAGCAGTTGGTTGTGC-3′) [37], and 5F2 (5′-GGGGWGAYCAGAAGAAGGC-3′) and 7cR (5′-CCCATRGCTTGYTTRCCCAT-3′) [38,39], respectively. The PCR amplifications were performed in a final volume of 12.5 μL: 6.25 μL of MyTaq MasterMix 2× (Bioline, Memphis, MN, USA), 0.3 μL (10 pmol/µL) of each primer, 4.25 μL of nuclease-free water, and 1 μL of template DNA (25 ng/μL). The cycling conditions were the following: initial denaturation at 95 °C for 1.5 min, followed by 35 cycles at 95 °C for 20 s, 53 °C (*tub*) for 45 s, 72 °C for 1 min, and a final extension for 5 min. Different annealing temperatures were used according to the different genomic regions amplified: 53 °C (*lsu-its*), 56 °C (*tef*), and 54 °C (*gapdh* and *rpb2*). PCR products were purified and bidirectionally Sanger-sequenced.

### 2.4. Phylogenetic Analyses

The DNA sequences and contig assembly of all isolates were checked for quality and ambiguity analysis through DNA Dragon software (v.1.9.2) (https://www.dna-dragon.com, accessed on 11 April 2025). A Bayesian Inference (BI) phylogenetic tree was initially reconstructed using the *tub* sequences of 75 isolates of this study. So, BLAST (v.2.17.0) searches were conducted on NCBI and Fusarioid-ID [40] for pairwise DNA alignments. Finally, a BI concatenate tree using *tub*, *tef*, *gapdh*, *lsu*, and *its* to *Setophoma* was reconstructed. *Didymella pinodella* CBS 531.66 was used as the outgroup. The alignments were made using Mega v.7 software [41], which were concatenated posteriorly, and the best nucleotide substitution models were determined for each genomic region with MrModeltest 2.3 [42]. The website CIPRES [43] was used to run MrBayes v 3.2.1 [44]. The Markov Chain Monte Carlo (MCMC) analysis was made under analysis of 10 million generations, sampling every 1000, with subsequent disposal of 25% first trees in the analysis, followed by the assembly of a consensus tree through the 7500 remaining trees of analysis with annotation of the posterior probability (PP) values. The consensus tree was visualized in FigTree v.1.4 [45].

### 2.5. Morphological Characterization of Setophoma terrestris

The morphological characterization of a representative *S. terrestris* isolate was performed using monohyphal culture grown on oatmeal agar (OA; 30 g/L oat and 15 g/L agar) medium on Petri dishes during seven days at 25 °C, under 24 h of near-ultraviolet light (UV-A or black light) to produce conidia and pycnidia. The morphological examination and documentation of pycnidia were observed and recorded by using a Leica model 205C stereomicroscope (Leica Microsystems, Nassloch, Germany) with a Leica DFC295 digital camera and Leica Qwin-Plus software (v.3.5). Micromorphological characteristics were analyzed by cutting pycnidia in a cryostat Leica CM1860 (Leica Microsystems, Nassloch, Germany) and mounting in clear lactoglycerol. Thirty-five measurements for conidia were carried out under magnification of ×1000 using a Leica DM2500 light microscope (Leica Microsystems, Nassloch, Germany) equipped with a Leica DFC 490 digital camera, coupled to a computer containing Leica Qwin-Plus software.

To induce the formation of chlamydospores and microsclerotia, six representative fungal isolates were grown in OA, synthetic low-nutrient agar (SNA; KH_2_PO_4_ 1 g/L, KNO_3_ 1 g/L, MgSO_4_.7H_2_O 0.5 g/L, KCl 0.5 g/L, glucose 0.2 g/L, sucrose 0.2 g/L, and agar 20 g/L), malt extract agar (MEA; 20 g/L malt extract and 20 g/L agar), PDA, PDB, corn meal agar (CMA; 50 g/L corn meal infused and 15 g/L agar), millet substrate, and modified yeast extract (MYE; sodium phosphate 2 g/L, magnesium sulfate 1 g/L, glucose 20 g/L, yeast extract 10 g/L). The isolates were incubated in the dark at 25 °C for 20 days. Subsequently, fungal structures were mounted on slides with clear lactoglycerol and analyzed under a Leica DM2500 light microscope (Leica Microsystems, Nassloch, Germany) equipped with a Leica DFC 490 digital camera, coupled to a computer containing Leica Qwin-Plus software.

### 2.6. Pathogenicity

Six representative isolates of *S. terrestris* (CCUB 2747, 2750, 2754, 2763, 2777, and 2795) selected based on region and host, and 25 isolates of *Fusarium*-like, identified by molecular phylogeny (*tub* tree), were used in pathogenicity tests on onion and garlic plants. Firstly, plastic bags with millet (500 g) and distilled water (100 mL) were previously autoclaved at 121 °C for 12 min. Twenty mycelial disks (5 mm in diameter) from colonies with 7-days-old in PDA were transferred to a sterilized millet substrate. After 30 days, when the millet had been completely colonized, 30 g were transferred to 3-liter pots containing sterilized soil, and then onion and garlic seedlings (30 days old) were transplanted near the inoculum. The inoculated genotypes were ‘Ito’ (garlic) and ‘BRS 367’ (onion). The control treatment consisted of 30 g of sterile millet. Finally, plants were removed after approximately 100 days and checked for pink-root symptoms and, subsequently, taken to fungi reisolation. The isolate exhibiting the highest aggressiveness on garlic and onion was selected, and a millet substrate was employed for pathogenicity assays on leeks, brachiaria, chives, and maize. Additionally, a second methodology was tested, where the seedlings suffered root cuts (~1 cm). Garlic (Ito) and onion (BRS 367) seedlings were carefully removed from the pots, and their roots were cut off using sterilized scissors. The roots were then immediately immersed in 0.5 L of a conidial suspension (1 × 10^4^ conidia/mL) for 10 min, and subsequently transferred to new 3-liter pots containing sterilized soil. The control treatment consisted of seedlings immersed in sterilized water.

## 3. Results

### 3.1. Phylogenetic Analysis

**Figure 1:**
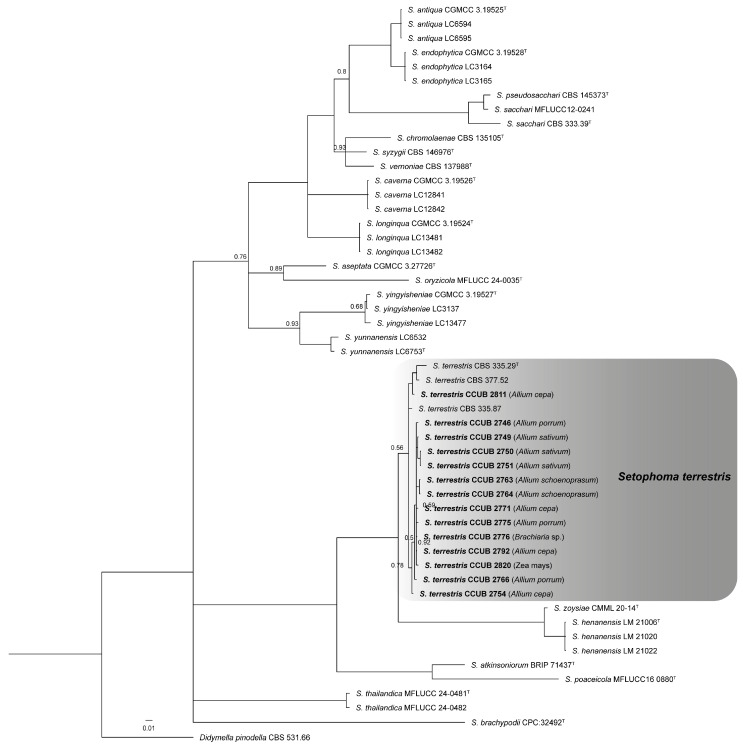
Bayesian phylogenetic tree based on concatenated sequences (*tub*, *tef*, *its*, *lsu*, and *gapdh*). Bayesian posterior probability (PP) values are indicated at the nodes, and the scale bar represents the number of expected changes per site (0.01). Branches without values indicate PP ≤ 0.95. The specimen *Didymella pinodella* CBS 531.66 was used as outgroup. The isolates obtained in this study were highlighted in bold. (^T^ = Type specimen).

After a BLAST search, it was possible to verify the presence of *Setophoma* sp. (*n* = 50) and *Fusarium* spp. (*n* = 25). The 12 selected isolates for multigenic analysis were compared to all *Setophoma* species (Supplementary Table S1). The *tub*, *tef*, *gapdh*, *its*, and *lsu* matrices had lengths, respectively, of 506, 575, 564, 603, and 837 bp. Although the *rpb2* sequences were not used in phylogenetic analysis, they were lodged in GenBank (Accession No. OM417590 to OM417601) for future studies and identification purposes. The concatenated alignment had 3085 sites, of which 2105 were conserved and 679 were parsimony informative. The BI tree was reconstructed considering the best nucleotide substitution model for each partition in the concatenated data, GTR + I + G (*tub*), GTR + G (*tef*), SYM + G (*gapdh*), HKY + G (*its*), and SYM + I (*lsu*). After multigenic analysis, it was confirmed that all isolates grouped to *S. terrestris* (Figure 1). These sequences were deposited in Genbank with the following codes: *tub* = ON159208 to ON159257; *tef* = ON159281 to ON159292; *gapdh* = ON159261 to ON159272, and *lsu-its* = OM397056 to OM397067.

### 3.2. Taxonomy

*Setophoma terrestris* (H.N. Hansen) Gruyter, Aveskamp and Verkley, Mycologia 102, 5, 1077 (2010) [GenBank: MB514659]

Basionym: *Phoma terrestris* H.N. Hansen, Phytopathology 19:699. 1929.

≡ *Pyrenochaeta terrestris* (H.N. Hansen) Gorenz, J.C. Walker and Larson, Phytopathology 38:838. 1948.

**Figure 2:**
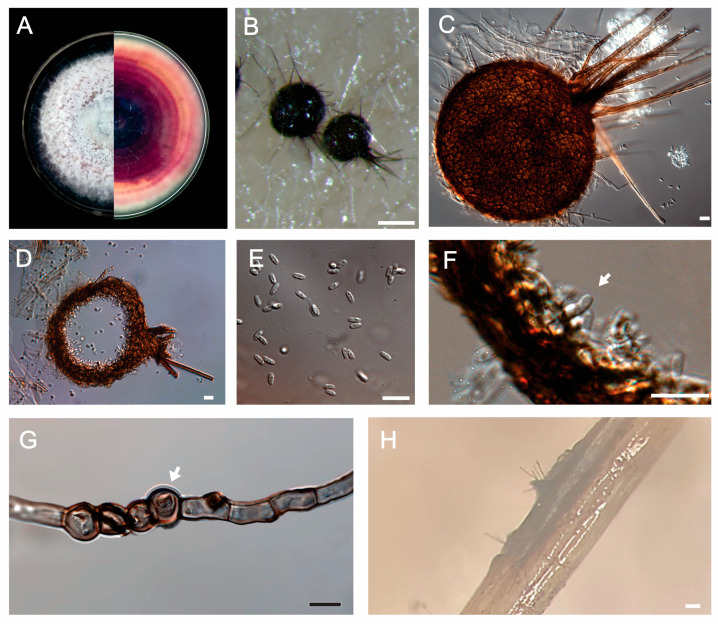
Morphological characteristics of *S. terrestris* CCUB 2754. (**A**): Culture grown on oatmeal agar (left, above; right, reverse); (**B**): setose pycnidia. Bar = 0.1 mm; (**C**,**D**): setose pycnidium and conidia. Bar = 10 µm; (**E**): conidia. Bar = 10 µm; (**F**): conidiogenous cell (arrow). Bar = 10 µm. (**G**): intercalar chlamydospores (arrow). Bar = 10 µm; (**H**): setose pycnidium on garlic root. Bar = 0.1 mm.

Pycnidial conidiomata are solitary to confluent, on the upper surface or submerged in agar, globose to subglobose, and setose, with papillate ostioles, and are medium to dark brown; they are the pycnidial wall of pseudoparenchymatal cells (Figure 2B–D,H). Conidiophores are reduced to conidiogenous cells lining the inner cavity (Figure 2F). Conidiogenous cells are hyaline, smooth, phialidic, and discrete. Aseptate conidia are globose, subglobose, ellipsoidal to subcylindrical to subfusoid, 4.5 to 6.0 µm in length (mean = 5.14 ± 0.45 µm), and 2.0 to 3.5 µm in width (mean = 2.56 ± 0.29 µm) (Figure 2E). Terminal and intercalar chlamydospores were observed in MYE (Figure 2G).

### 3.3. Pathogenicity Test

**Figure 3:**
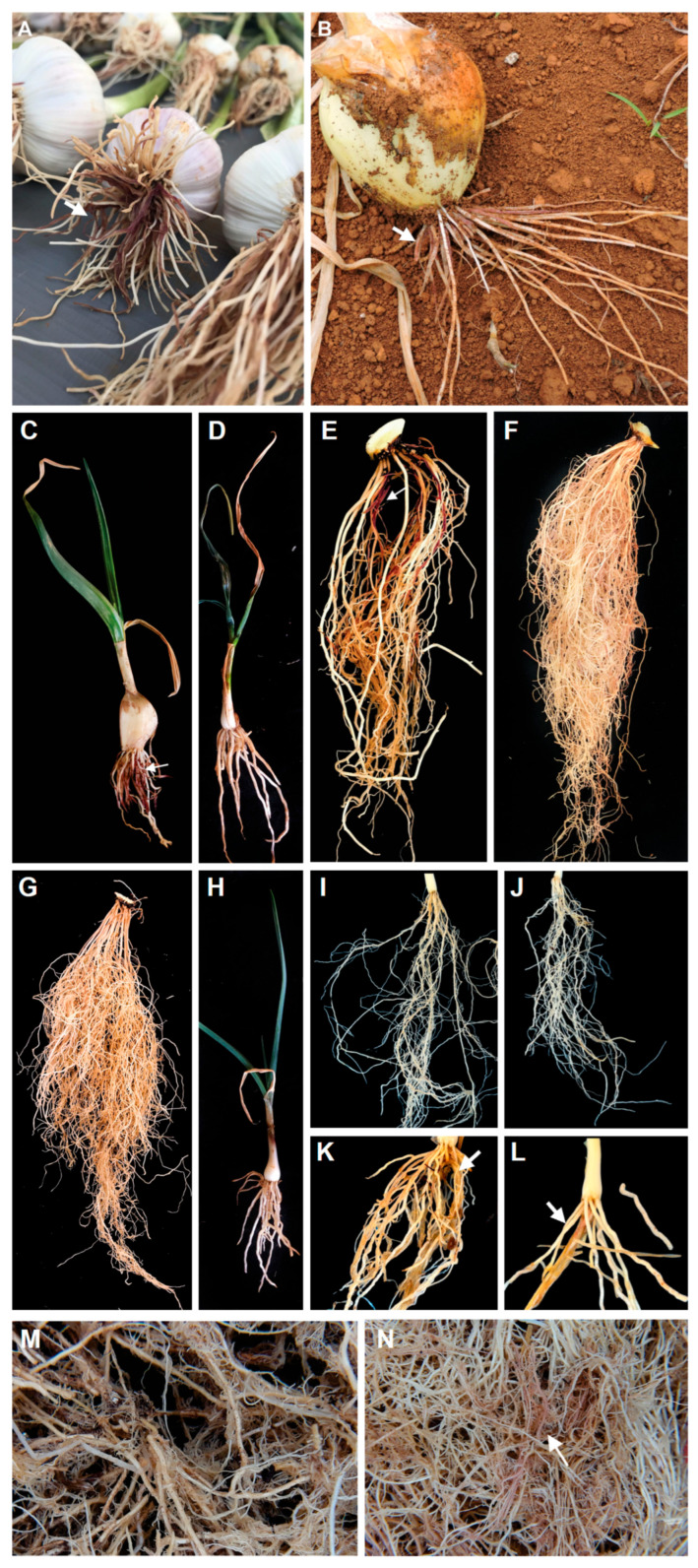
Pink root (arrows) symptoms under field and greenhouse conditions. Garlic (**A**) and onion (**B**) showing symptoms in field; garlic (**C**), onion (**E**), chives (**K**), leeks (**L**) and brachiaria (**N**) inoculated with *S. terrestris* isolate CCUB 2754 under greenhouse conditions; garlic (**D**), onion (**F**), chives (**I**), leeks (**J**), and brachiaria (**M**) mock-inoculated controls; garlic (**G**) and onion (**H**) asyntomatic after inoculation with *Fusarium* sp.

The six representative isolates of *S. terrestris* were pathogenic to onion and garlic in millet substrate and spore suspension (Figure 3). Disease visualization was more consistent when using the millet substrate, whereas the method involving root cuts immersed in spore suspensions resulted in some asymptomatic roots. Differently, plants inoculated with the 25 *Fusarium* isolates were asymptomatic in both methodologies. Thus, *S. terrestris* was confirmed as the only causal agent of pink root disease in Brazil. In addition, the *S. terrestris* isolate CCUB2754 was pathogenic to leeks, brachiaria, chives, and maize.

## 4. Discussion

Although *Setophoma* and *Fusarium* are found in leaves, soil, and the rhizosphere, the highest abundance is observed in roots [4]. In Brazil, the incidence of pink root is lower in regions with mild climates (South and Southeast) compared to regions with high temperatures (Midwest). Furthermore, the disease gradually increases in areas with successive crops and can drastically increase in plantations during the summer due to high temperatures combined with periods of drought stress [4,17,46,47].

All 25 *Fusarium* isolates obtained in this study were considered asymptomatic on onion and garlic roots, as no visible lesions or discoloration were observed following inoculation. Although non-pathogenic *Fusarium* species are frequently associated with pink root symptoms [17,19,24], some species are known to cause Fusarium basal rot [48]. Conversely, *Setophoma* isolates caused characteristic light to dark pink discoloration on the roots of brachiaria, chives, garlic, leeks, maize, and onion (Figure 3). In our study, only light to dark pink discoloration of infected roots was observed, which is likely attributable to the controlled experimental conditions and the timing of the assessments prior to bulb harvest. In contrast, field soils harbor an active microbiota and are frequently exposed to high temperatures combined with prolonged water stress, which may exacerbate symptom severity under natural conditions [4,5,46,49].

In this study, the *Setophoma* isolates (*n* = 50) collected in the states of Bahia, Goiás, and Distrito Federal (Midwest and Northeast), and Minas Gerais, Paraná, São Paulo, and Santa Catarina (South and Southeast) were obtained from symptomatic tissues on *Allium cepa* (*n* = 26), *A. porrum* (*n* = 4), *A. sativum* (*n* = 9), *A. fistulosum* (*n* = 7), *Brachiaria* sp. (*n* = 2), and *Zea mays* (*n* = 2). *Setophoma terrestris* colonizes canola, carrot, cauliflower, bunching onion, corn, cowpea, cucumber, eggplant, elephant garlic, eschalote, leeks, lima bean, melon, oats, pea, pepper, potato, pumpkin, sorghum, soybean, spinach, sugarcane, rice, tomato, wheat, and weeds [3,17,50,51,52,53,54,55,56,57,58]. Nevertheless, only maize, garlic, onion, pea, and tomato were previously recorded as hosts of this fungus in Brazil [58,59]. To our knowledge, this is the first report of *S. terrestris* associated with pink root in brachiaria, chives, and leeks in Brazil.

The clade assignments based on the concatenated-sequence tree (*lsu*, *its*, *tef*, *tub*, and *gapdh*) confirm twenty *Setophoma* species (Figure 1). There is a proposal to split *Setophoma* into three genera, with *Setophoma* stricto sensus including *S. terrestris*, *S. brachypodii*, and *S. poaceicola* [60]. Large-scale studies that investigate the pink root etiology are scarce in the literature. Although several *Setophoma* species have been reported on *Camellia* in China [29], concatenated analysis (*lsu*, *its*, *tef*, *tub*, and *gapdh*) of isolates obtained from different geographic regions and hosts confirmed only *S. terrestris* causing pink root. The absence of cryptic species in *S. terrestris* is likely, given its role as a component of the soil microbiome and its lack of host specificity. Additionally, this fungus exhibits potentially antagonistic interactions with other soil organisms and is known to produce bioactive secondary metabolites, including cytotoxic polyketides [61,62].

Some studies report that *S. terrestris* survives in the soil due to the formation of chlamydospores and microsclerotia [17,28]. The presence of chlamydospores and microsclerotia was not observed in symptomatic plants and in pathogenicity tests. After isolation in pure culture, we attempted to induce the formation of these structures on OA, SNA, MEA, CMA, PDA, PDB, millet substrate, and MYE. While microsclerotia were not observed under the experimental conditions of this study, chlamydospores were observed only on MYE. Efficient use of the supplied nitrogen source during early somatic growth is essential, while subsequent nutrient depletion induces physiological stress that promotes chlamydospore formation [63]. Several studies on *S. terrestris* did not mention the presence of these structures [49,61]. In addition, among all taxonomic studies of *Setophoma*, only the description of *S. thailandica* reported the presence of chlamydospores, whereas microsclerotia have not been observed in any study [27,29,64,65,66,67,68,69,70]. Therefore, the main sources of primary inoculum of pink root disease are chlamydospores, pycnidia, colonized roots of garlic, onion, and plant debris of susceptible crops.

## 5. Conclusions

The confirmation that only *S. terrestris* causes pink root in garlic and onion in Brazil, and the absence of cryptic species associated with this pathogen, is essential for validating disease diagnostic protocols. Its presence in the roots of brachiaria, chives, leeks, maize, and other hosts demonstrates the ability of this fungus to survive in alternative plants during the interseason. This new information will be fundamental for breeders and plant pathologists in the development of garlic and onion genotypes resistant to pink root, and will contribute to more efficient disease management in the field.

## Data Availability

The original contributions presented in this study are included in the article/Supplementary Materials. Further inquiries can be directed to the corresponding authors.

## Supplementary Materials

The following supporting information can be downloaded at: https://www.mdpi.com/article/10.3390/jof11080581/s1, Table S1: GenBank accession numbers of *tub*, *lsu*, *its*, *tef*, and *gapdh* and *rpb2* partial sequences of *Setophoma* isolates included in this study.

## References

[1] FAO—Food and Agriculture Organization. 2024. Faostats-FAO Statistical Databases. https://www.fao.org/faostat/en/#data.

[2] Reis A., Oliveira V.R., Lourenço Junior V. (2016). Identificação e Manejo Da Raiz Rosada Da Cebola.

[3] Sumner D.R., Schwartz H.F., Mohan K. (2008). Pink Root. Compendium of Onion and Garlic Diseases and Pests.

[4] Gao C., Montoya L., Xu L., Madera M., Hollingsworth J., Purdom E., Singan V., Vogel J., Hutmacher R.B., Dahlberg J.A. (2020). Fungal community assembly in drought-stressed sorghum show stochasticity, selection, and universal ecological dynamics. Nat. Commun..

[5] Sadeghi S., Nasr-Esfahani M., Maleki M., Molahoseini H., Khankahdani H.H., Mohammadi M. (2024). Fungicidal control of onion pink root caused by *Setophoma terrestris* and effects on soil enzyme activity. J. Phytopathol..

[6] Sayago P., Juncosa F., Albarracín Orio A.G., Luna D.F., Molina G., Lafi J., Ducasse D.A. (2020). *Bacillus subtilis* ALBA01 alleviates onion pink root by antagonizing the pathogen *Setophoma terrestris* and allowing physiological status maintenance. Eur. J. Plant Pathol..

[7] Taubenhaus J.J., Johnson A.D. (1917). Pink Root, a New Root Disease of Onions in Texas. Phytopathology.

[8] Chaves G.M., Erickson H.T. (1960). *Pyrenochaeta terrestris* (Hansen) Gorenz, J.C. Walker y Larson, Um Novo Fungo Da Cebola (*Allium cepa* L.) Em Minas Gerais. Rev. Ceres.

[9] Luz N.L. (1967). Raízes Rosadas-Uma Nova Moléstia Da Cebola Para o Rio Grande Do Sul. Rev. Da Fac. De. Agron. E Veterinária Da Univ. Fed. Do Rio Gd. Do Sul..

[10] Noda H. (1981). Reação Da Cebola (Allium cepa L.) a Pyrenochaeta terrestris (Hansen), Gorenz, Walker e Larson, Escola Superior de Agricultura “Luiz de Queiroz”.

[11] Zhao P., Feng Z., Cai L., Phurbu D., Duan W., Xie F., Li X., Liu F. (2024). Development of an RPA-CRISPR/Cas12a Assay for Rapid and Sensitive Diagnosis of Plant Quarantine Fungus *Setophoma terrestris*. J. Fungi.

[12] Zhang F.B., Zheng H.L., Cui W.G., Zhang M.Q., Yin Y.S., Cui M., Gao M. (2019). First report of *Setophoma terrestris* causing pink root of garlic in China. Plant Dis..

[13] Taubenhaus J.J., Mally F.W. (1921). Pink Root Disease of Onions and Its Control in Texas.

[14] Taubenhaus J.J. (1919). Pink Root of Onions. Science.

[15] Sideris C.P. (1924). Species of *Fusarium* Isolated from Onion Roots. Phytopathology.

[16] Sideris C.P. (1929). The effect of the H-ion concentration of the culture solution on the behaviour of *Fusarium cromyophthoron* and *Allium cepa* and the development of pink-root disease symptoms. Phytopathology.

[17] Yoshida N. (2022). Seasonal Dynamics of the Pink Root Fungus (*Setophoma terrestris*) in Rhizosphere Soil: Effect of Crop Species and Rotation. Plant Pathol..

[18] Hansen H.N. (1926). “Pink-Root” Of Onions Caused By *Phoma* sp.. Science.

[19] Hansen H.N. (1929). Etiology of the Pink Root Disease of Onions. Phytopathology.

[20] Du Plessis S.J. (1933). Pink Root and Bulbrot of Onions.

[21] Tims E.C. (1953). Pink Root of Shallots, *Allium ascalonicum*. Plant Dis. Rep..

[22] Watson R.D. (1961). Rapid Identification of the Onion Pink Root Fungus. Plant Dis. Rep..

[23] Kodama F., Sugawara Y., Yokoyama T. (1976). Pink Root Rot of Onion Caused by *Pyrenochaeta terrestris*. Phytopathol. Soc. Jpn..

[24] Awuah R.T., Lorbeer J.W. (1989). A Procedure for Isolating *Pyrenochaeta terrestris* from Onion Roots. Ann. Appl. Biol..

[25] Gorenz A.M., Larson R.H., Walker J.C. (1949). Factors Affecting Pathogenicity of Pink Root Fungus of Onions. J. Agric. Res..

[26] Gorenz A.M., Walker J.C., Lardon R.H. (1948). Morphology and Taxonomy of the Onion Pink-Root Fungus. Phytopathology.

[27] de Gruyter J., Woudenberg J.H.C., Aveskamp M.M., Verkley G.J.M., Groenewald J.Z., Crous P.W. (2010). Systematic Reappraisal of Species in *Phoma* Section *Paraphoma*, *Pyrenochaeta* and *Pleurophoma*. Mycologia.

[28] Biles C.L., Holland M., Ulloa-Godinez M., Clason D., Corgan J. (1992). *Pyrenochaeta terrestris* Microsclerotia Production and Pigmentation on Onion Roots. HortScience.

[29] Liu F., Wang J., Li H., Wang W., Cai L. (2019). *Setophoma* spp. on *Camellia sinensis*. Fungal Syst. Evol..

[30] Li X., Liu F., Duan W.J. (2024). TaqMan MGB-based real-time fluorescent PCR method for the rapid detection of *Setophoma terrestris*. Mycosystema.

[31] Alfenas A.C., Ferreira F.A., Mafia R.G., Gonçalves R.C., Alfenas A.C., Mafia R.G. (2016). Métodos Em Fitopatologia. Métodos em Fitopatologia.

[32] Pinho D.B., Firmino A.L., Ferreira-Junior W.G., Pereira O.L. (2013). An Efficient Protocol for DNA Extraction from *Meliolales* and the Description of *Meliola Centellae* sp. nov. Mycotaxon.

[33] O’Donnell K., Cigelnik E. (1997). Two Divergent Intragenomic RDNA ITS2 Types within a Monophyletic Lineage of the Fungus *Fusarium* Are Nonorthologous. Mol. Phylogenet. Evol..

[34] Vilgalys R., Hester M. (1990). Rapid Genetic Identification and Mapping of Enzymatically Amplified Ribosomal DNA from Several *Cryptococcus* Species. J. Bacteriol..

[35] de Hoog G.S., van den Ende A.H.G.G. (1998). Molecular Diagnostics of Clinical Strains of Filamentous Basidiomycetes. Mycoses.

[36] Jacobs K., Bergdahl D.R., Wingfield M.J., Halik S., Seifert K.A., Bright D.E., Wingfield B.D. (2004). *Leptographium wingfieldii* Introduced into North America and Found Associated with Exotic *Tomicus piniperda* and Native Bark Beetles. Mycol. Res..

[37] Berbee M.L., Pirseyedi M., Hubbard S. (1999). *Cochliobolus* Phylogenetics and the Origin of Known, Highly Virulent Pathogens, Inferred from ITS and Glyceraldehyde-3-Phosphate Dehydrogenase Gene Sequences. Mycologia.

[38] Liu Y.J., Whelen S., Hall B.D. (1999). Phylogenetic Relationships among Ascomycetes: Evidence from an RNA Polymerse II Subunit. Mol. Biol. Evol..

[39] Sung G.-H., Sung J.-M., Hywel-Jones N.L., Spatafora J.W. (2007). A Multi-Gene Phylogeny of Clavicipitaceae (Ascomycota, Fungi): Identification of Localized Incongruence Using a Combinational Bootstrap Approach. Mol. Phylogenet Evol..

[40] Crous P.W., Lombard L., Sandoval-Denis M., Seifert K.A., Schroers H.-J., Chaverri P., Gené J., Guarro J., Hirooka Y., Bensch K. (2021). *Fusarium*: More than a Node or a Foot-Shaped Basal Cell. Stud. Mycol..

[41] Kumar S., Stecher G., Tamura K. (2016). MEGA7: Molecular Evolutionary Genetics Analysis Version 7.0 for Bigger Datasets. Mol. Biol. Evol..

[42] Nylander J.A.A. (2004). MrModeltest Version 2. Program Distributed by the Author.

[43] Miller M.A., Pfeiffer W., Schwartz T. (2010). Creating the CIPRES Science Gateway for Inference of Large Phylogenetic Trees. Proceedings of the 2010 Gateway Computing Environments Workshop (GCE).

[44] Ronquist F., Huelsenbeck J.P. (2003). MrBayes 3: Bayesian Phylogenetic Inference under Mixed Models. Bioinformatics.

[45] Rambaut A. (2018). FigTree. http://tree.bio.ed.ac.uk/software/figtree/.

[46] Abd-El-Baky A.A., Yousef H., Shalaby S.I. (2019). Garlic pink rot disease and crop yield as affected by salinity and irrigation water deficit. Egypt. J. Phytopathol..

[47] Ferreira J.F., Bosland P.W., Williams P.H. (1991). The variability of *Pyrenochaeta terrestris* isolates based on isozyme polymorphism, cultural characteristics and virulence on differential onion breeding lines. J. Phytopathol..

[48] Le D., Audenaert K., Haesaert G. (2021). Fusarium basal rot: Profile of an increasingly important disease in *Allium* spp.. Trop. Plant Pathol..

[49] Nico A.I., Sánchez M.G. (2012). Response of Different Intermediate-Day Onion Hybrids to Natural Infestation by *Phoma terrestris* and *Fusarium oxysporum* f. sp. *cepae* in Ciudad Real, Spain with Assessment of Different Soil Disinfestation Methods. Eur. J. Plant Pathol..

[50] Crous P.W., Phillips A.J.L., Baxter A.P. (2000). Phytopathogenic Fungi from South Africa.

[51] López-López A.M., Tovar-Pedraza J.M., León-Félix J., Allende-Molar R., Bernardi Lima N., Márquez-Zequera I., García-Estrada R.S. (2024). Caracterización morfológica, filogenia y patogenicidad de *Setophoma terrestris* causante de raíz corchosa y rosada de jitomate (*Solanum lycopersicum*) en Sinaloa, México. Rev. Mex. Fitopatol..

[52] Babadoost M. Onion Pink Root: Report on Plant Disease. 1990. University of Illinois Extension RPD No. 932. https://ipm.illinois.edu/diseases/rpds/932.pdf.

[53] Ikeda K., Kuwabara K., Urushibara T., Soyai P., Miki S., Shibata S. (2012). Pink Root Rot of Squash Caused by *Setophoma terrestris* in Japan. J. Gen. General. Plant Pathol..

[54] Levic J., Petrovic T., Stankovic S., Ivanovic D. (2013). The Incidence of *Pyrenochaeta terrestris* in Root of Different Plant Species in Serbia. Zb Matice Srp Prir Nauk.

[55] Punithalingram E., Holiday P. (1973). Descriptions of Fungi and Bacteria. https://www.cabidigitallibrary.org/doi/10.1079/DFB/20056400397.

[56] Yang Y., Zuzak K., Harding M., Neilson E., Feindel D., Feng J. (2017). First Report of Pink Root Rot Caused by *Setophoma (Pyrenochaeta) terrestris* on Canola. Can. J. Plant Pathol..

[57] López-López M., Léon-Félix J., Allende-Molar R., Lima N.B., Tovar-Pedraza J.M., García-Estrada R.S. (2020). First Report of *Setophoma terrestris* Causing Corky and Pink Root of Tomato in Sinaloa, Mexico. Plant Dis..

[58] (2024). USDA Fungal Databases. https://fungi.ars.usda.gov/.

[59] Wordell Filho J.A., Rowe E., Gonçalves P.A.S., Debarba J.F., Boff P., Thomazelli L.F., Wordell Filho J.A., Boff P. (2006). Manejo Fitossanitário Na Cultura Da Cebola. Doenças de Origem Parasitária.

[60] Ferreira B.W., Guterres D.C., Macedo D.M., Barreto R. (2023). Epityfication of *Perisporiopsis struthanthi* and *Perisporiopsis lantanae*, and the taxonomic implications for Perisporiopsidaceae, *Perisporiopsis* and *Setophoma*. ResearchSquare.

[61] Rivera-Méndez W., Brenes-Madriz J., Alvarado-Marchena L. (2021). Effect of *Setophoma terrestris*, *Sclerotium cepivorum*, and *Trichoderma* spp. on in Vitro Onion (*Allium cepa*) Root Tissues and the Final Yield at the Field. Eur. J. Plant Pathol..

[62] El-Elimat T., Figueroa M., Raja H.A., Graf T.N., Swanson S.M., Falkinham J.O., Oberlies N.H. (2015). Biosynthetically distinct cytotoxic polyketides from *Setophoma terrestris*. Eur. J. Org. Chem..

[63] Peng X., Wu B., Zhang S., Li M., Jiang X. (2021). Transcriptome dynamics underlying chlamydospore formation in *Trichoderma virens* GV29-8. Front. Microbiol..

[64] Marin-Felix Y., Hernández-Restrepo M., Iturrieta-González I., García D., Gené J., Groenewald J.Z., Cai L., Chen Q., Quaedvlieg W., Schumacher R.K. (2019). Genera of Phytopathogenic Fungi: GOPHY 3. Stud. Mycol..

[65] Phookamsak R., Liu J.-K., Manamgoda D.S., Chukeatirote E., Mortimer P.E., Mckenzie E.H.C., Hyde K.D. (2014). The Sexual State of *Setophoma*. Phytotaxa.

[66] Yasanthika W.A.E., Chethana K.W.T., Fatimah A.-O., Wanasinghe D.N., Hyde K.D. (2025). Genera of soil Ascomycota and an account on soil-inhabiting species isolated from Thailand. Mycosphere.

[67] Cai Y.-T., Zhang L., Shen H.-W., Bao D.-F., Luo Z.-L. (2025). *Setophoma aseptata* sp. nov. and new record of *Minutisphaera aspera* from Yuanjiang River Basin, China. Phytotaxa.

[68] Absalan S., Armand A., Jayawardena R.S., McKenzie E.H.C., Hyde K.D., Lumyong S. (2024). Diversity of Pleosporalean Fungi Isolated from Rice (*Oryza sativa* L.) in Northern Thailand and Descriptions of Five New Species. J. Fungi.

[69] Li M., Liu D., Wang M., Cui K., Chen L., He L., Zhou L. (2025). Characterization of *Setophoma henanensis* sp. nov., causing root rot on peanut. BMC Microbiol..

[70] Liu H.F., Choi H.J., Paul N.C., Ariyawansa H.A., Sang H.K. (2025). 2025. Discovering fungal communities in roots of *Zoysia japonica* and characterising novel species and their antifungal activities. IMA Fungus.

