# Supplementary material for: The Role of Setophoma terrestris in Pink Root Disease: New Insights and Host Range in Brazil
Source: J Fungi (Basel). 2025 Aug 5;11(8):581. doi: 10.3390/jof11080581 (PMC12387970; doi:10.3390/jof11080581)
Supplement: Supplementary file 1 [file jof-11-00581-s001.zip › jof-3758159-supplementary.pdf]

Table S1. GenBank accession numbers of *tub, lsu, its, tef, gapdh* e *rpb2* partial sequences of *Setophoma* isolates included in this study.

		GenBank accession numbers							
**Species**	**Strain**	***tub***	***lsu***	***its***	***tef***	***gapdh***	***rpb2***	**Host**	**Locality**
Didymella pinodella	CBS 531.66	FJ427162	GU238017	FJ427052	MK525067	MK532379		*Trifolium pretense*	USA
S. antiqua	LC6594	MK524999	MK511947	MK511909	MK525070	MK525034		*Camellia sinensis*, pathogen	Yunnan, China
S. antiqua	LC6595	MK525000	MK511948	MK511910	MK525071	MK525035		*Camellia sinensis*, pathogen	Yunnan, China
S. antiqua	CGMCC 3.19525 ^{T}	MK525001	-	MK511911	MK525072	MK525036		*Camellia sinensis*, pathogen	Yunnan, China
S. aseptata	CGMCC 3.27726^{T}	-	PQ626034	PQ625993	-	-			China
S. atkinsoniorum	BRIP 71437 ^{T}	-	OK349509	OK349508	-	-			
S. brachypodii	CPC:32492^{T}	-	-	NR_164462	MK540161	-		*Brachypodium sylvaticum*	Belgium
S. chromolaenae	CBS 135105 ^{T}	KF252728	KF251747	KF251244	KF253195	-		*Chromolaena odorata*	Brazil
S. endophytica	CGMCC 3.19528 ^{T}	MK525020	MK511956	MK511931	MK525092	MK525053		*Camellia sinensis*, endophyte	Jiangxi, China
S. endophytica	LC3164	MK525021	MK511957	MK511932	MK525093	MK525054		*Camellia sinensis*, endophyte	Jiangxi, China
S. endophytica	LC3165	MK525022	-	MK511933	MK525094	MK525055		*Camellia sinensis*, endophyte	Jiangxi, China
S. henanensis	LM 21006^{T}	OQ834258	OQ819327	OQ819329	OQ834254	-		*Arachis hypogaea*	China
S. henanensis	LM 21022	OQ834256	OQ819325	OQ819330	OQ834253	-		*Arachis hypogaea*	China
S. henanensis	LM 21020	OQ834257	OQ819326	OQ819328	OQ834255	-		*Arachis hypogaea*	China
S. longinqua	CGMCC 3.19524^{T}	MK524998	MK511946	MK511908	MK525069	-		*Camellia sinensis*, pathogen	Yunnan, China
S. longinqua	LC13481	MK525014	-	MK511925	MK525086	-		*Camellia sinensis*, pathogen	Yunnan, China
S. longinqua	LC13482	MK525015	-	MK511926	MK525087	-		*Camellia sinensis*, pathogen	Yunnan, China
S. oryzicola	MFLUCC 24-0035^{T}	-	PQ376623	PQ376620	-	-		*Oryza sativa*	Thailand
S. poaceicola	MFLUCC 16-0880^{T}	-	KY550386	KY568988	-	-			Thailand
S. pseudosacchari	CBS 145373^{T}	MK540176	-	MK539969	-	-		-	-
S. sacchari	CBS 333.39^{T}	-	NG_057837	NR_145173	-	-		*Saccharum officinarum*	Brazil
S. sacchari	MFLUCC 12-0241	-	KJ476147	-	-	-		*Saccharum officinarum*	Thailand

S. syzygii	CBS 146976^{T}	MZ078272	MZ064503	MZ064446	-	-			
S. terrestris	CBS 335.29^{T}	KF252729	KF251749	KF251246	KF253196	-		*Allium sativum*	USA
S. terrestris	CBS 335.87	KF252730	KF251750	KF251247	KF253197	-		*Allium cepa*	Senegal
S. terrestris	CBS 377.52	KF252731	KF251751	KF251248	KF253198	-		*Allium cepa*	-
S. terrestris	**CCUB 2746**	ON159208	-	-	-	-		*Allium porrum*	Taguatinga, DF, Brazil
S. terrestris	**CCUB 2747**	ON159209	-	-	-	-		*Allium porrum*	Sobradinho, DF, Brazil
S. terrestris	**CCUB 2749**	ON159210	OM397056	OM397056	ON159281	ON159261	OM417590	*Allium sativum*	Cristalina, GO, Brazil
S. terrestris	**CCUB 2750**	ON159211	OM397057	OM397058	ON159282	ON159262	OM417591	*Allium sativum*	Cristalina, GO, Brazil
S. terrestris	**CCUB 2751**	ON159212	OM397058	OM397058	ON159283	ON159263	OM417592	*Allium sativum*	Cristalina, GO, Brazil
S. terrestris	**CCUB 2753**	ON159213	-	-	-	-		*Allium sativum*	Cristalina, GO, Brazil
S. terrestris	**CCUB 2754**	ON159214	OM397059	OM397059	ON159284	ON159264	OM417593	*Allium cepa*	São Gotardo, MG, Brazil
S. terrestris	**CCUB 2755**	ON159215	-	-	-	-		*Allium cepa*	Rio Paranaíba, MG, Brazil
S. terrestris	**CCUB 2756**	ON159216	-	-	-	-		*Allium cepa*	Rio Paranaíba, MG, Brazil
S. terrestris	**CCUB 2757**	ON159217	-	-	-	-		*Allium cepa*	Cristalina, GO, Brazil
S. terrestris	**CCUB 2758**	ON159218	-	-	-	-		*Allium cepa*	Cristalina, GO, Brazil
S. terrestris	**CCUB 2760**	ON159219	-	-	-	-		*Allium schoenoprasum*	Vargem Bonita, DF, Brazil
S. terrestris	**CCUB 2761**	ON159220	-	-	-	-		*Allium schoenoprasum*	Vargem Bonita, DF, Brazil
S. terrestris	**CCUB 2763**	ON159221	OM397060	OM397060	ON159285	ON159265	OM417594	*Allium schoenoprasum*	Brazlândia, DF, Brazil
S. terrestris	**CCUB 2764**	ON159222	OM397061	OM397061	ON159286	ON159266	OM417595	*Allium schoenoprasum*	Brazlândia, DF, Brazil
S. terrestris	**CCUB 2765**	ON159223	-	-	-	-		*Allium porrum*	Brazlândia, DF, Brazil
S. terrestris	**CCUB 2766**	ON159224	OM397062	OM397062	ON159287	ON159267	OM417596	*Allium sativum*	São Gotardo, MG, Brazil
S. terrestris	**CCUB 2769**	ON159225	-	-	-	-		*Allium schoenoprasum*	Guarapuava, PR, Brazil
S. terrestris	**CCUB 2770**	ON159226	-	-	-	-		*Allium sativum*	Cristalina, GO, Brazil
S. terrestris	**CCUB 2771**	ON159227	OM397063	OM397063	ON159288	ON159268	OM417597	*Allium cepa*	Cristalina, GO, Brazil
S. terrestris	**CCUB 2772**	ON159228	-	-	-	-		*Allium sativum*	Rio Paranaíba, MG, Brazil

S. terrestris	**CCUB 2775**	ON159229	-	-	-	-		*Allium porrum*	Cristalina, GO, Brazil
S. terrestris	**CCUB 2776**	ON159230	OM397064	OM397064	ON159289	ON159269	OM417598	*Brachiaria* sp.	Cristalina, GO, Brazil
S. terrestris	**CCUB 2777**	ON159231	-	-	-	-		*Brachiaria* sp.	Cristalina, GO, Brazil
S. terrestris	**CCUB 2779**	ON159232	-	-	-	-		*Allium cepa*	Palmital, MG, Brazil
S. terrestris	**CCUB 2780**	ON159233	-	-	-	-		*Allium cepa*	Palmital, MG, Brazil
S. terrestris	**CCUB 2789**	ON159234	-	-	-	-		*Allium cepa*	Cabeceira Grande, MG, Brazil
S. terrestris	**CCUB 2791**	ON159235	-	-	-	-		*Allium cepa*	João Dourado, BA, Brazil
S. terrestris	**CCUB 2792**	ON159236	OM397065	OM397065	ON159290	ON159270	OM417599	*Allium cepa*	Irecê, BA, Brazil
S. terrestris	**CCUB 2795**	ON159237	-	-	-	-		*Allium schoenoprasum*	Sobradinho, DF, Brazil
S. terrestris	**CCUB 2797**	ON159238	-	-	-	-		*Allium schoenoprasum*	Sobradinho, DF, Brazil
S. terrestris	**CCUB 2798**	ON159239	-	-	-	-		*Allium cepa*	Contenda, PR, Brazil
S. terrestris	**CCUB 2802**	ON159240	-	-	-	-		*Allium cepa*	Irecê, BA, Brazil
S. terrestris	**CCUB 2805**	ON159241	-	-	-	-		*Allium cepa*	Atalanta, SC, Brazil
S. terrestris	**CCUB 2806**	ON159242	-	-	-	-		*Allium cepa*	Contenda, PR, Brazil
S. terrestris	**CCUB 2808**	ON159243	-	-	-	-		*Allium cepa*	Guarapuava, PR, Brazil
S. terrestris	**CCUB 2809**	ON159244	-	-	-	-		*Allium cepa*	Guarapuava, PR, Brazil
S. terrestris	**CCUB 2811**	ON159245	OM397066	OM397066	ON159291	ON159271	OM417600	*Allium cepa*	Guarapuava, PR, Brazil
S. terrestris	**CCUB 2812**	ON159246	-	-	-	-		*Allium cepa*	Guarapuava, PR, Brazil
S. terrestris	**CCUB 2813**	ON159247	-	-	-	-		*Allium cepa*	Guarapuava, PR, Brazil
S. terrestris	**CCUB 2815**	ON159248	-	-	-	-		*Allium cepa*	Imbuía, SC, Brazil
S. terrestris	**CCUB 2816**	ON159249	-	-	-	-		*Allium cepa*	Imbuía, SC, Brazil
S. terrestris	**CCUB 2817**	ON159250	-	-	-	-		*Allium cepa*	Imbuía, SC, Brazil
S. terrestris	**CCUB 2820**	ON159251	OM397067	OM397067	ON159292	ON159272	OM417601	*Zea mays*	Serra Bonita, MG, Brazil
S. terrestris	**CCUB 2823**	ON159252	-	-	-	-		*Allium sativum*	São Gotardo, MG, Brazil
S. terrestris	**CCUB 2826**	ON159253	-	-	-	-		*Allium cepa*	Palmital, MG, Brazil

S. terrestris	**CCUB 2827**	ON159254	-	-	-	-	*Allium cepa*	Batatais, SP, Brazil
S. terrestris	**CCUB 2830**	ON159255	-	-	-	-	*Allium sativum*	Brazlândia, DF, Brazil
S. terrestris	**CCUB 2832**	ON159256	-	-	-	-	*Allium cepa*	João Dourado, BA
S. terrestris	**CCUB 3353**	ON159257	-	-	-	-	*Zea mays*	Cristalina, GO, Brazil
S. thailandica	MFLUCC 24-0481[T]	-	PQ577781	PP993943	-	-	Soil	Thailand
S. thailandica	MFLUCC 24-0482	-	-	PQ577782	-	-	Soil	Thailand
S. vernoniae	CBS 137988[T]	MK540177	-	KJ869141	MK540162	-	*Vernonia polyanthes*	Viçosa, MG, Brazil
S. yingyisheniae	LC13477	MK525005	MK511952	MK511916	MK525077	MK525040	*Camellia sinensis*, pathogen	Fujian, China
S. yingyisheniae	CGMCC 3.19527[T]	MK525007	-	MK511918	MK525079	MK525042	*Camellia sinensis*, pathogen	Guangxi, China
S. yingyisheniae	LC3137	MK525019	-	MK511930	MK525091	MK525052	*Camellia sinensis*, pathogen	Jiangxi, China
S. caverna	LC12841	MK525016	-	MK511927	MK525088	MK525049	Carbonatite in cave	Guizhou, China
S. caverna	LC12842	MK525017	-	MK511928	MK525089	MK525050	Carbonatite in cave	Guizhou, China
S. caverna	CGMCC 3.19526 [T]	MK525032	MK511965	MK511944	MK525105	MK525066	Carbonatite in cave	Guizhou, China
S. yunnanensis	LC6753[T]	MK525003	-	-	MK525074	-	*Camellia sinensis*	China
S. yunnanensis	LC6532	MK524997	-	-	MK525068	-	*Camellia sinensis*	China
S. zoysiae	CMML 20-14 [T]	-	PQ741507	PQ741482	-	-		South Korea

[T] =Type specimen
